# Influence of patient motion on definition of typical cephalometric reference points in digital horizontally scanning cephalometric radiography

**DOI:** 10.1186/s13005-025-00491-z

**Published:** 2025-03-17

**Authors:** Kim Martin, Christos Katsaros, Robert Brylka, Ulrich Schwanecke, Ralf Schulze

**Affiliations:** 1 Department of Oral Surgery and Stomatology and Oral Diagnostics, School of Dental Medicine, University of Bern, Freiburgstrasse 7, Bern, 3010 Switzerland; 2 Department of Orthodontics and Dentofacial Orthopedics, School of Dental Medicine, University of Bern, Freiburgstrasse 7, Bern, 3010 Switzerland; 3 Computer Vision and Mixed Reality Group, RheinMain University of Applied Sciences Wiesbaden Rüsselsheim, Wiesbaden, 65197 Germany; 4 Division of Oral Diagnostic Sciences, School of Dental Medicine, University of Bern, Freiburgstrasse 7, Bern, 3010 Switzerland

**Keywords:** Head movement, Diagnostic imaging, Digital cephalometric radiography, Cephalometric analysis, Scanning technique

## Abstract

**Background:**

The aim of this study was to investigate the effect of defined head-motion during x-ray exposure on the identification accuracy of typical cephalometric reference points.

**Methods:**

By means of a dry adult human skull and a precise motion simulation system digital Cephs are acquired while certain predefined movement patterns (shift, tilt and nodding with a motion amplitude from 5 – 50 mm) of the skull were executed. They represent the movements of children and adolescents, the main group for cephalometric radiographs.The scanning time was 9.4 s per Ceph. 10 typical landmark points of cephalometric analysis were identified by 20 observers on each Ceph twice. Using a non-motion image (Ceph0) as reference, displacement was computed as vectors relative to this image. Commonly used angles and vertical and horizontal distances were calculated.

**Results:**

Both inter-rater as well as intra-rater-reproducibility were perfect. There was very little change in the vertical distance N-Me, in contrast to the horizontal distance S–N which showed a large variation. So patient motion parallel to the scanning direction of the fan-beam-detector unit, heavily influence distances parallel to this direction. The ANB angle and the Maxillo-Mandibular Plane Angle (ANS-PNS to Me-Go) only varied by about 1–2°.

**Conclusions:**

The study observed a severe influence on reference point location of motion patterns parallel to the scanning direction and also on clinically relevant distances parallel to the scanning direction. Therefore, we recommend to use a horizontal scanning direction, to minimise scanning time to a minimum, or to prefer a one-shot technique if possible. Future advancements in this field may include the integration of artificial intelligence or algorithms for the purpose of motion correction.

**Supplementary Information:**

The online version contains supplementary material available at 10.1186/s13005-025-00491-z.

## Introduction

Digital cephalometric radiographs (Ceph) are acquired with a digital sensor. As sensor area is very costly, two methods have been implemented: a) demagnification of the Ceph to project it onto a small-size sensor, leaving no time for head movement or b) by using a vertically or horizontal scanning line-detector that is moved across the image-area [1, 2]. However, in some countries, such as Switzerland, only one-shot Cephs are reimbursed by insurance companies, as the acquisition time for scanned counterparts is much longer, increasing the risk of patient movement [3]. Scan times vary between 5 s and ca. 20 s for the scanning devices [3, 4], giving the patient enough time to move their head. Unfortunately, there are not many studies published on the motion patterns and amplitudes of patients during cephalometric image acquisition. In a video-controlled simulation study [2], Huh and colleagues observed generally larger movements with longer (simulated) exposure times. They found movement amplitudes of approx. 15 mm with long exposure times (20 s), but also often 1 mm to 5 mm with shorter exposure times. Motion was particularly prominent in the young age group (9 to 12 yrs) [2]. These data are in good agreement with those of Menzel and Gebauer [3]. The maximum they found was a vertical movement of around 17 mm [3]. The fact that patient movement in an X-ray device increases with decreasing age is also known for CBCT [5]. As Cephs are commonly acquired in young patients between 9 and 16 years, this age-dependency will have a considerable effect in Ceph-imaging [6].

There is no research so far investigating how orthodontic cephalometric analysis is affected by inaccuracies of cephalometric reference points due to movement. The aim of this study was therefore to investigate the influence of a defined head movement on the recognition accuracy of typical cephalometric reference points. By means of a human skull, a highly accurate motion control platform, and multiple observers, the accuracy of reference-point definitions shall be evaluated and the influence of head-motion analysed.

## Methods

### X-ray device and motion simulation system

**Fig. 1:**
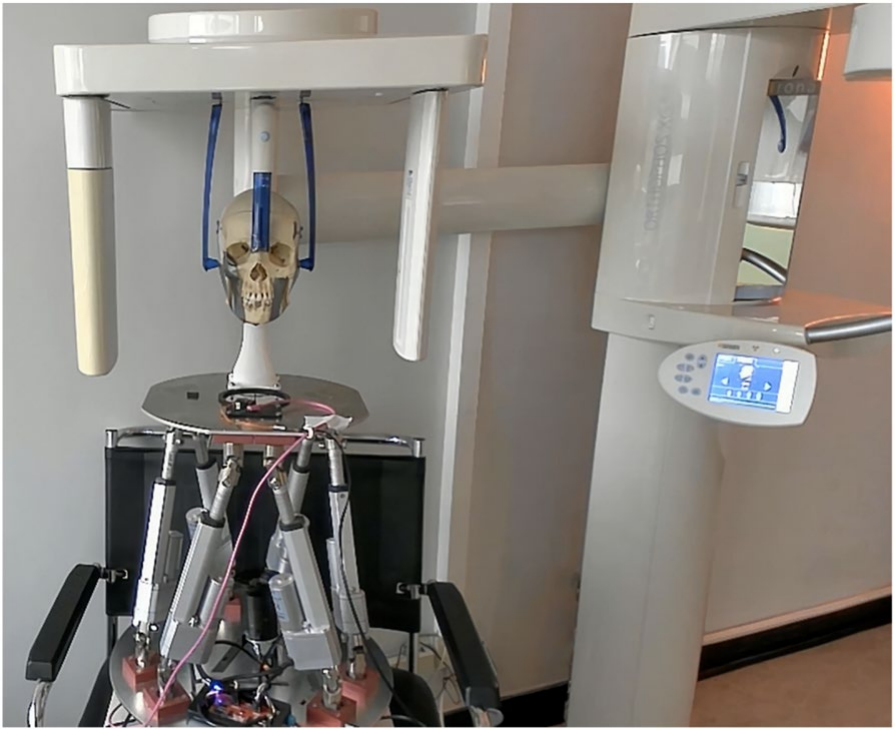
Head movement simulation system with mounted dry skull prepared for x-ray acquisition

A dry adult human skull was used as object for this investigation. It was mounted on a head motion simulation system allowing predefined movements. It is a commercially available Stewart platform (https://acrome.net/products/stewart-pro), which we have extended and adapted for our purposes (Details in supplementary materials). Cephs were acquired in the Orthophos XG plus DS/Ceph (Sirona Dental Systems GmbH, Bensheim, Germany; exact specifications see Table 1 in [7]). The vertical fan-beam is moved horizontally from anterior to posterior through the acquisition area. Consequently, the Ceph-image is acquired sequentially from the patient’s forehead to the back. The acquisition time for this study was 9.4 s per Ceph. Figure 1 shows our system with the dry skull mounted and prepared for recording.

### Motion patterns

We applied different patterns and magnitudes for this evaluation, using the results of Menzel and Gebauer [3] as a guide, but also extending them to get a better overview.

The patterns included forward and backward motion in the sagittal plane, vertical nodding, and high-frequency tremor. Patterns are detailed in supplementary materials.

**Fig. 2:**
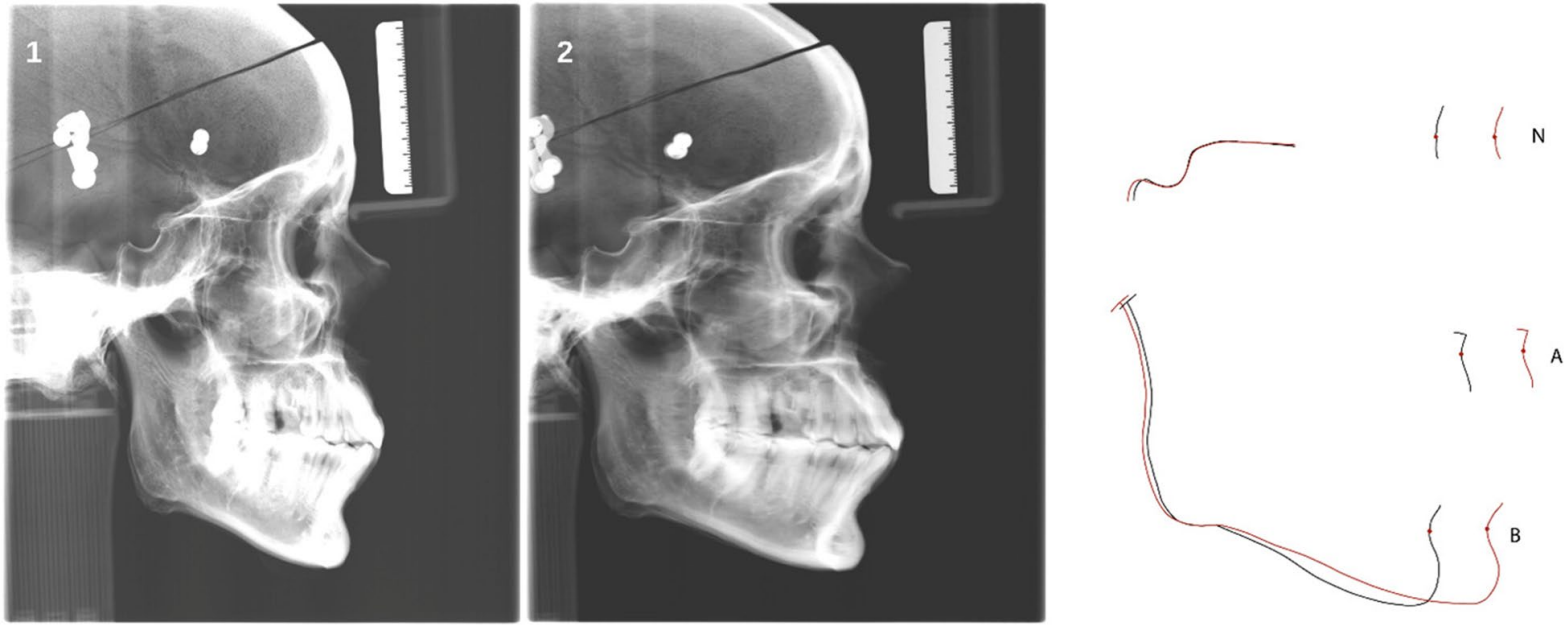
Reference Ceph0 (1) versus Ceph No. 2 (2), where with a dorsal shift of 20 mm was applied. (3) Superimposition of the outlines of the two radiographs (black: Ceph0, red: Ceph No. 2) on the anterior cranial base, showing the large anterior displacement of the points N, A and B as a result of the dorsal shift

For each Ceph (example: Fig. 2), at the beginning, the skull was reset to its initial position, so that the start-position of the skull relative to the image receptor and the X-ray tube was identical for all images. A non-motion Ceph (Ceph0) was acquired from the skull serving as reference image. It turned out that Ceph No. 3 with a dorsal shift of 10 cm rendered unreadable and was thus omitted from the sample.

### Cephalometric analysis

**Fig. 3:**
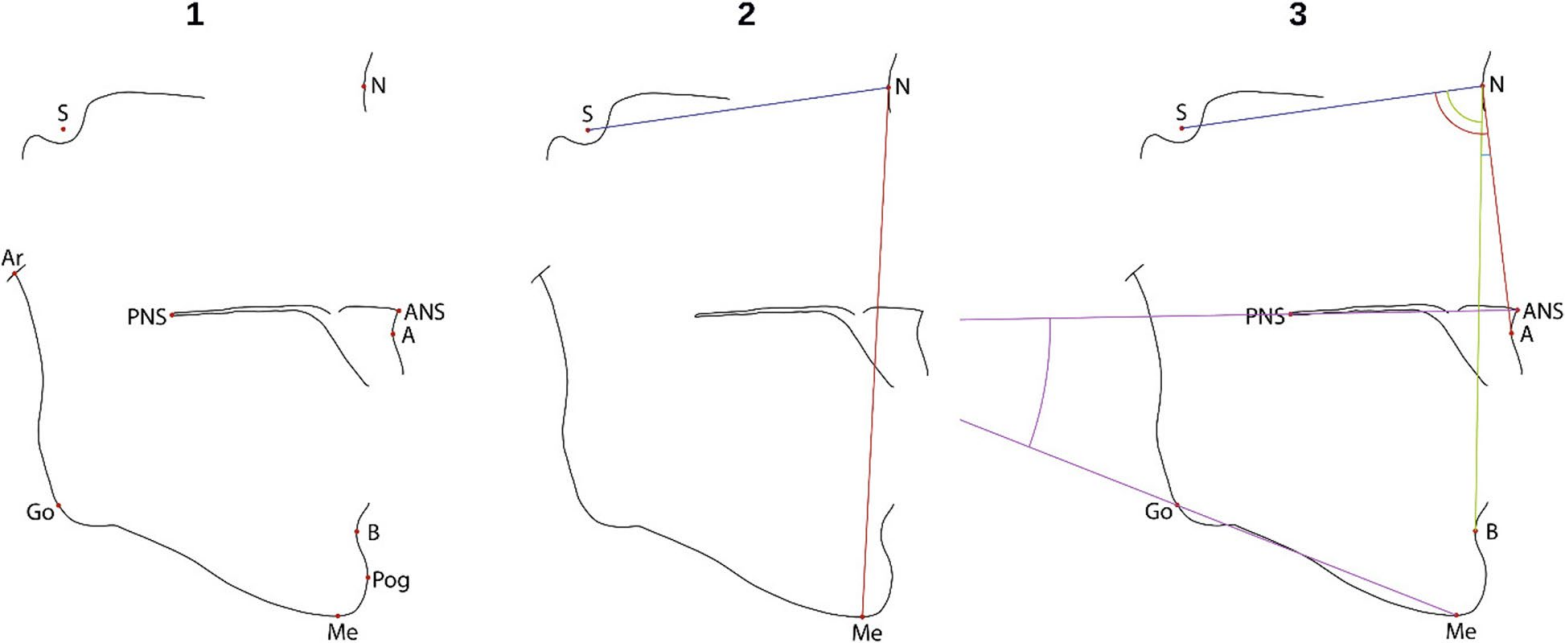
Cephalometric points (left), distances (middle) and angles (right) used in this study, marked on the skull’s outlines. The definition of the points can be found in the supplemental information

A total of 10 landmarks were defined as ‘typical’ landmark points in the sense that they are commonly used in cephalometric analysis. The selected landmark points are pictured in Fig. 3. They are specified in the supplementary materials.

A total of 20 observers were asked to identify the landmark points on each Ceph twice, with a time interval of at least four weeks. These were opened in ImageJ [8] and displayed in 1:1 mode (one image pixel is displayed on one monitor pixel) in a quiet and darkened room on a computer monitor (HP E273q, 2560 × 1440 pixel, pixel pitch 0.23 mm, HP Inc., Palo Alto, USA).

### Statistical analysis

The entire analysis was conducted in R [9] and the libraries ”psych” and ”irr”. Plots were entirely created by means of the library “ggplot2”. Displacement of the reference points as well as the distances and angles derived from these displacements were the primary outcome of this study. Using the non-motion image (Ceph No. 18) as reference, displacement of the reference points was computed as vectors (marked in bold letters) relative to this image. All following equations are required to compute these data.

Using the standard for digital images, the upper left corner of each Ceph was considered as origin (0,0). The distances $D_x$ and $D_y$ between their mean (over all observers) reference point location *A* with coordinates $A_x$ and $A_y$ in the respective Ceph *n* and the reference Ceph0 (No. 18) were computed from:

1 $${D}_{xn}=\text{mean }{x}_{{\text{Ceph}}_{\text{n}}}-\text{mean }{x}_{{\text{Ceph}}_{18}},{D}_{yn}=\text{mean }{y}_{{\text{Ceph}}_{\text{n}}}-\text{mean }{y}_{{\text{Ceph}}_{18}}$$

where the mean is computed over all observations of the 20 observers for Ceph n. For every Ceph (1 to 17) the length of the displacement vector **DA**_n_ between a reference point (here: A) in Ceph No. n and the reference image Ceph0 is given by:

2 $$\left|{\mathbf{D}\mathbf{A}}_{\text{n}}\right|=\sqrt{{{\mathbf{D}\mathbf{A}}_{\text{xn}}}^{2}+{{\mathbf{D}\mathbf{A}}_{\text{yn}}}^{2}}$$

Typical vertical (e.g. **NMe**) and horizontal (e.g. **SN**) distances between reference points were computed analogously. In addition, the angles SNB (and ANSPNS-MeGo) were calculated from (here exemplarily for SNB):3

where ⋆ is the dot-product and the lengths of the vectors are obtained from application of Eq. 2.

Absolute values and vector directions were related to motion-patterns and amplitudes.

Inter-rater and intra-rater reproducibility was computed by means of the intraclass coefficient (ICC) using a 2-way random effects model using both R-libraries ‘irrNA’ and ‘psych’. To assess potential differences in the vertical (y-) and horizontal (x-) direction, the ICC was computed for both coordinates separately.

## Results

Both inter-rater (ICC_x_ = 0.9990248, ICC_y_ = 0.9995254) as well as intra-rater-reproducibility (ICC_x_ = 0.9996143, ICC_y_ = 0.9997370) were perfect for both x- and y-coordinates. This is also indicated by a mean intra-observer difference (observation No 1-observation No 2) of 0.55 pixel (median = 0 pixel) for the x-coordinate versus a mean difference of 0.41 pixel (median = 0 pixel) for the y-coordinate. However, we observed a severe influence of motion patterns on the location of the respective reference points.

**Fig. 4:**
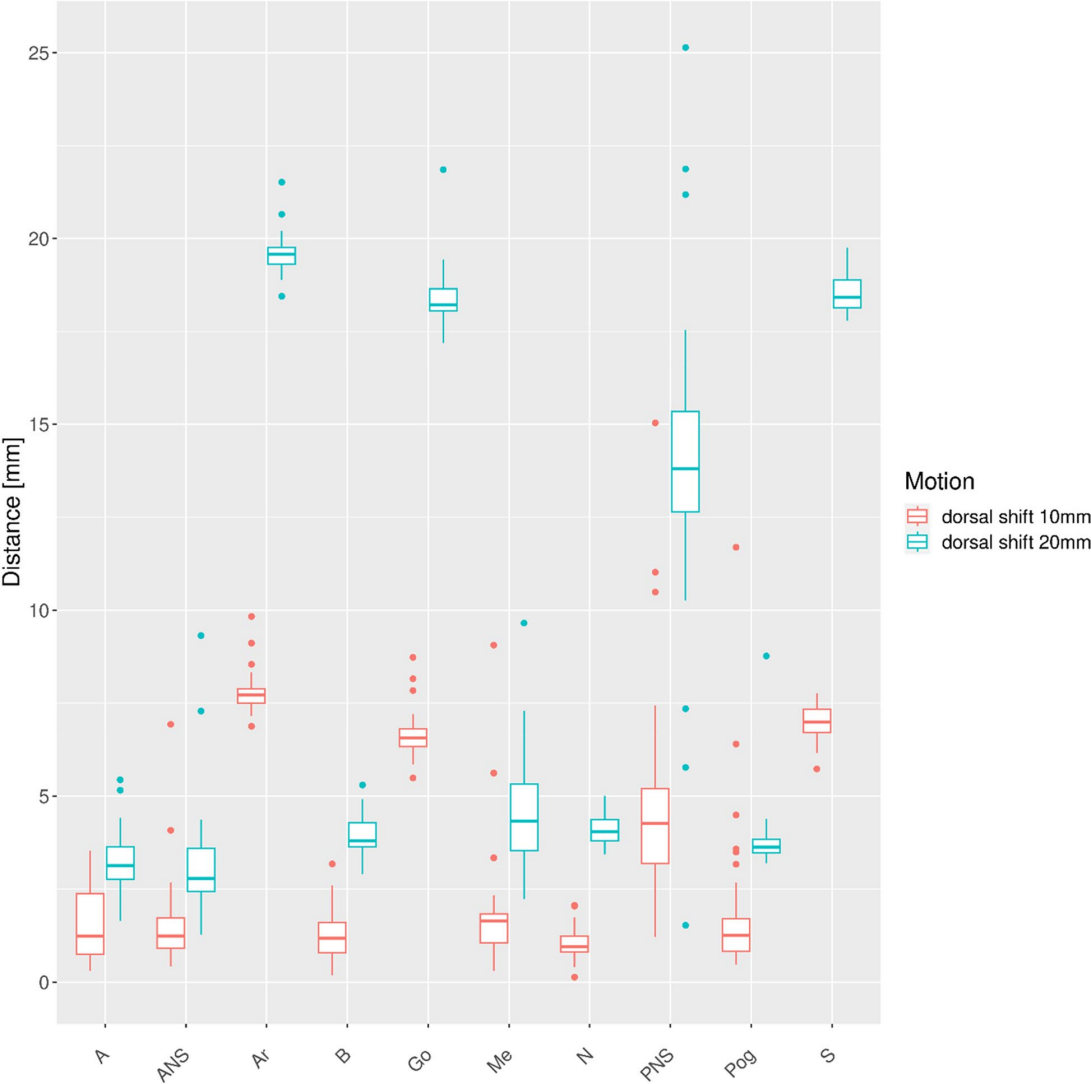
Deviations to points in reference Ceph0 for dorsal motion

**Fig. 5:**
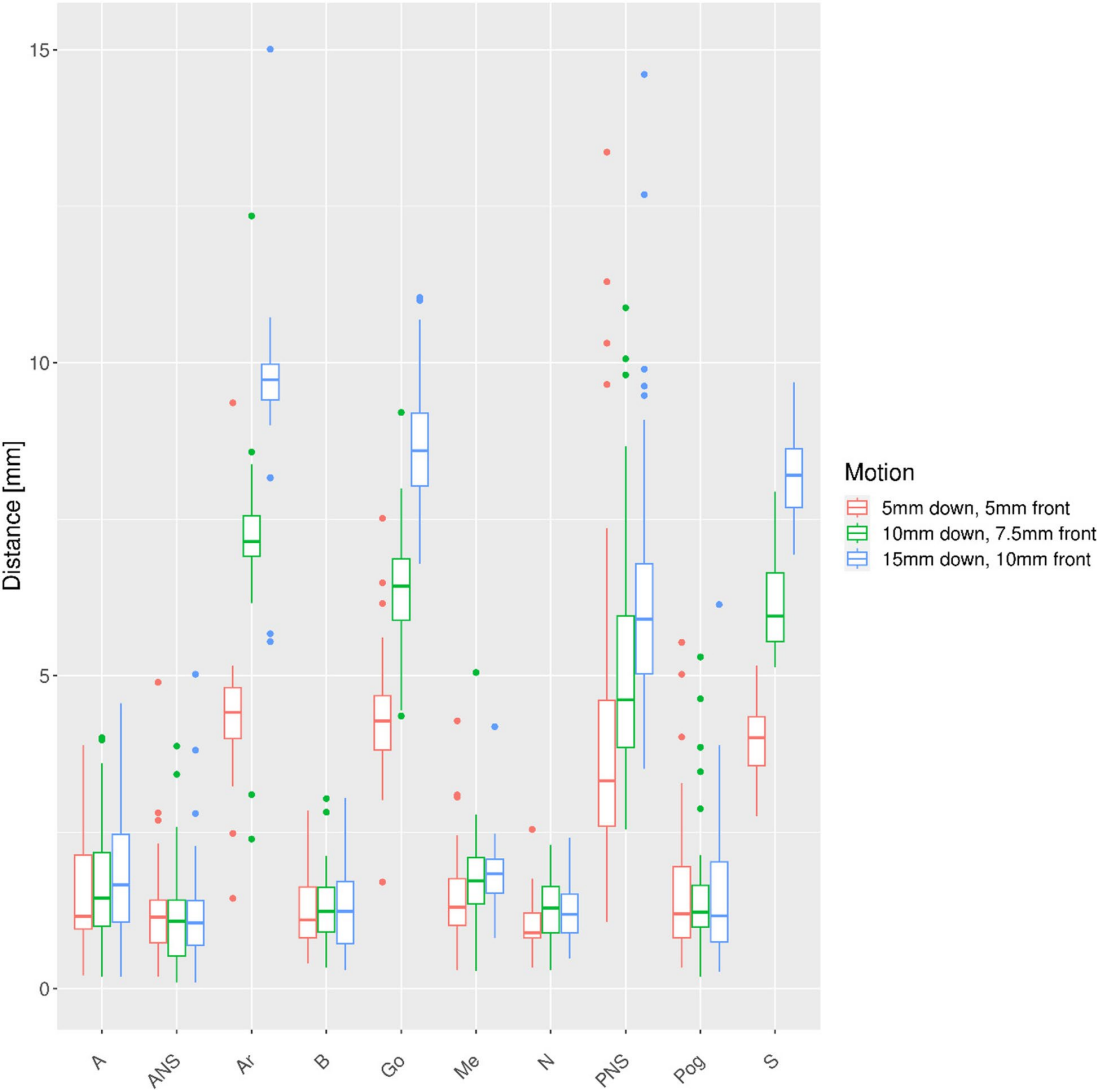
Deviations to points in reference Ceph0 for combined downward and frontal motion

**Fig. 6:**
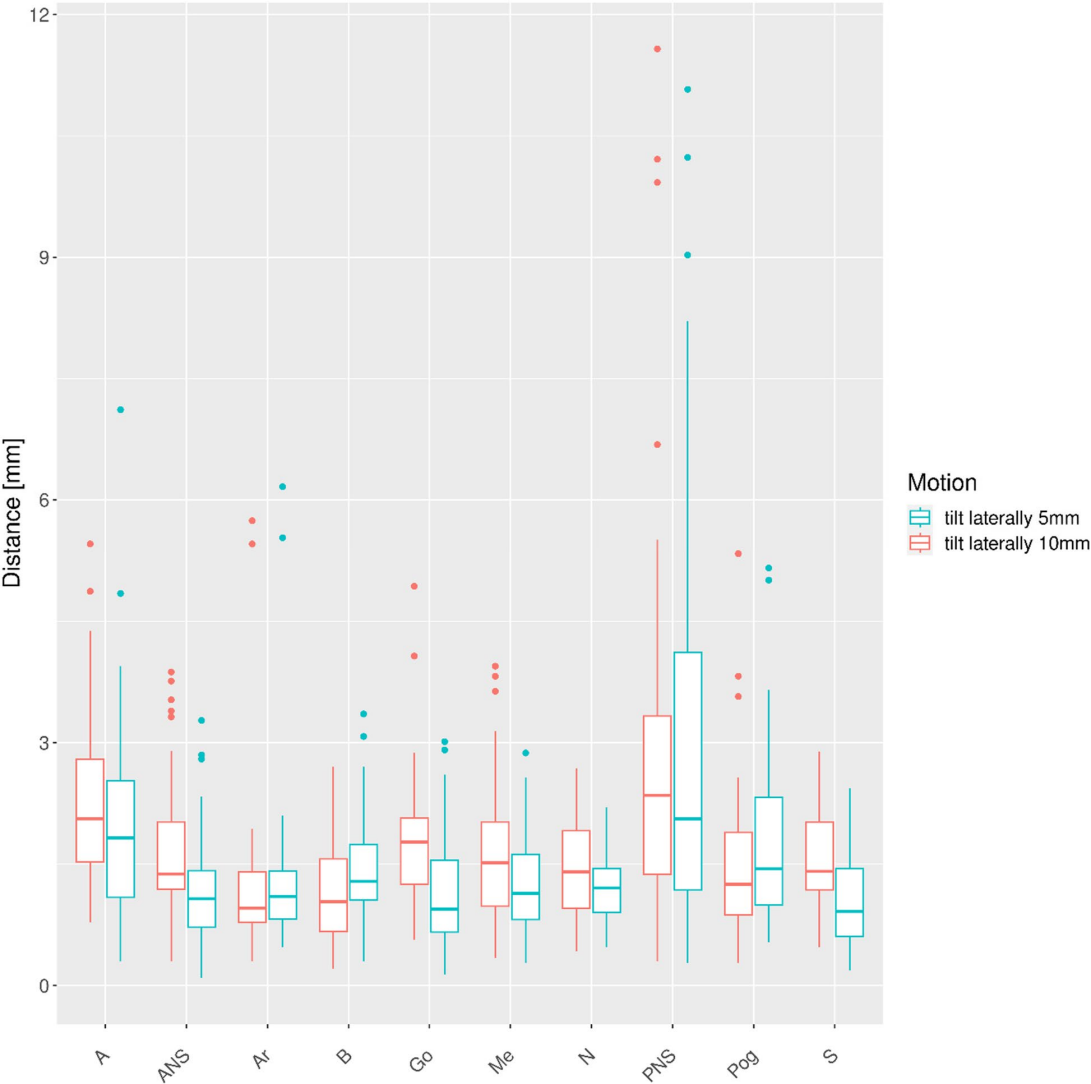
Distances to points in reference Ceph0 for lateral tilt of the head

**Fig. 7:**
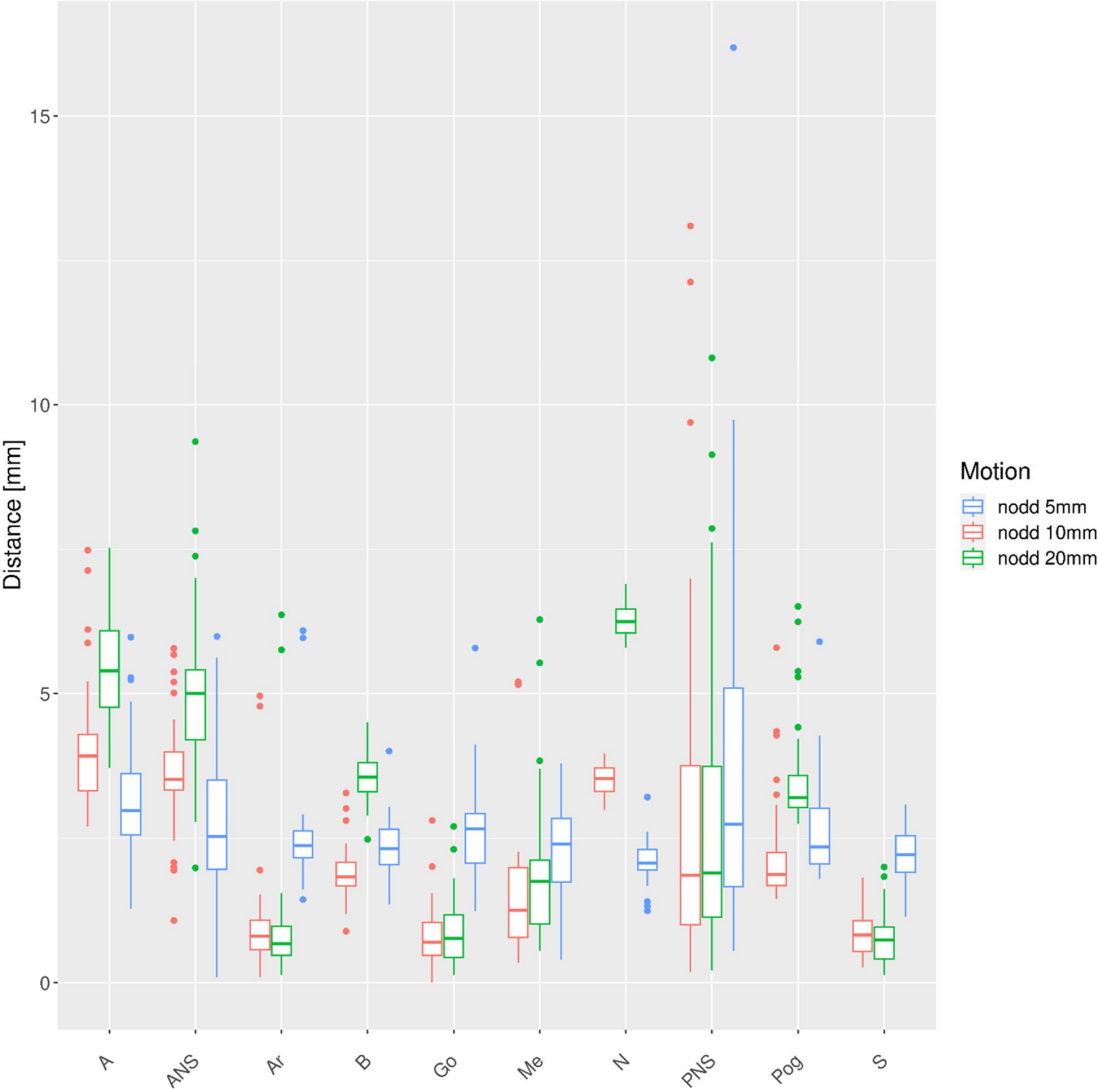
Deviations to points in reference Ceph0 for single nodding motion

**Fig. 8:**
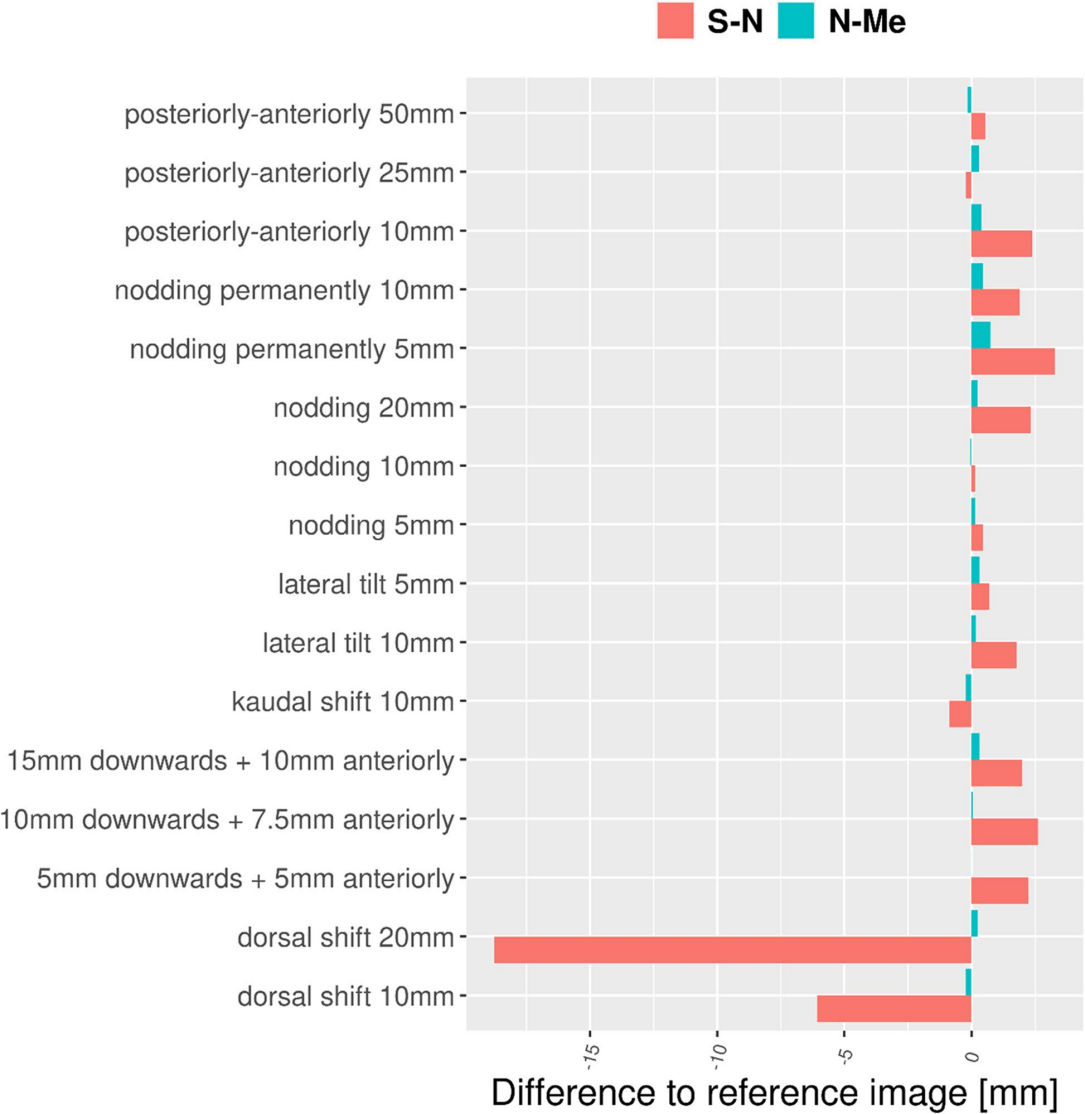
Influence of motion patterns on horizontal (S–N) versus vertical (N-Me) distances

**Fig. 9:**
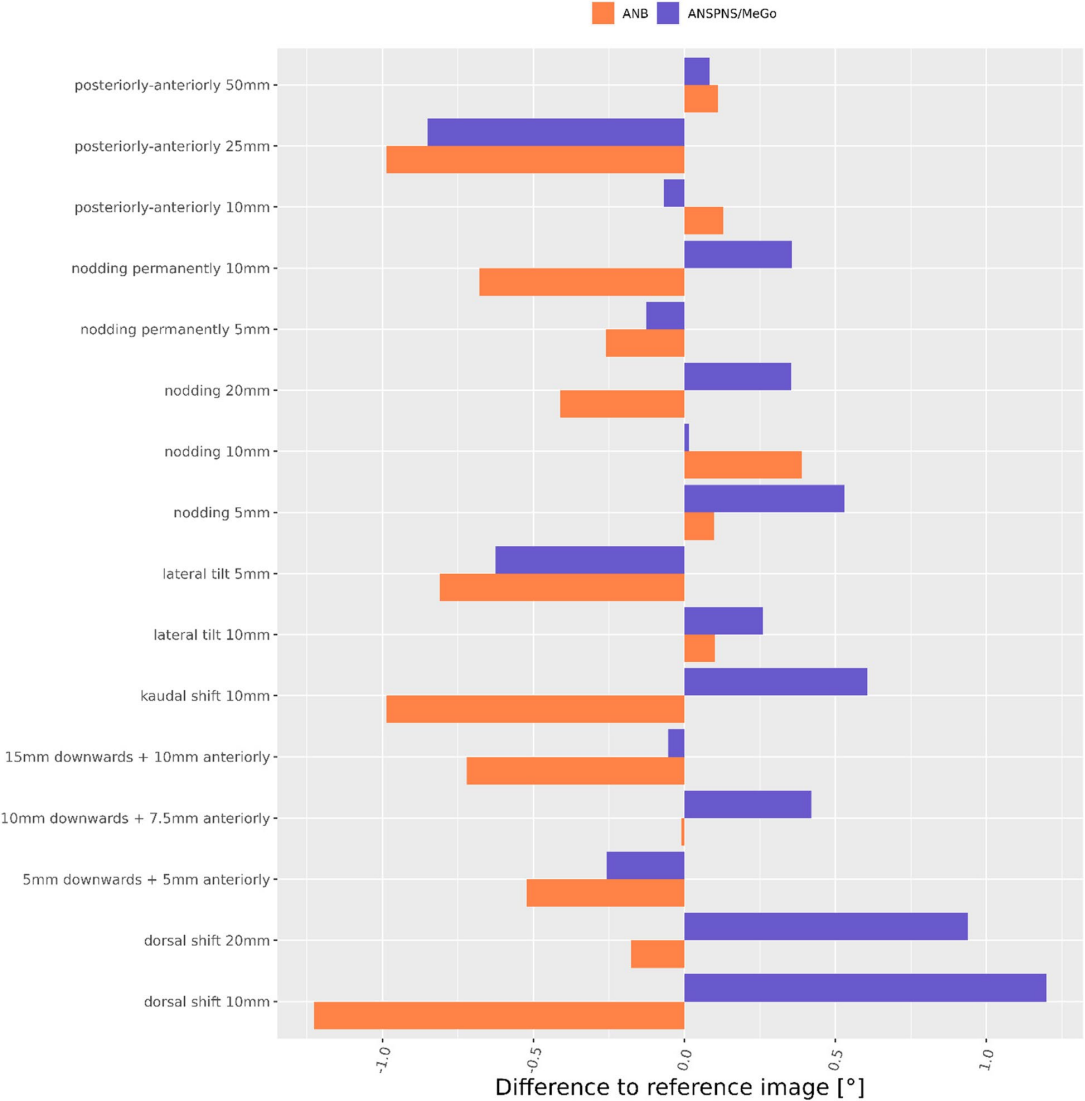
Angular deviations from reference image Ceph0 for ANB- and ANSPNS-MeGo-angle

The distances of the respective points relative to the Ceph0 are plotted for the different motion patters (Figs. 4, 5, 6, 7, 8 and 9). As expected, a dorsal shift in direction of the scanning motion caused the largest point deviations up to 25 mm (Fig. 4).

The vertical distance N-Me exhibited minimal deviations (range: 111.7 mm to 112.7 mm, mean = 112.3 mm) whereas the horizontal distance S–N (range: 60.8 mm to 82.8 mm, mean = 64.4 mm) (Fig. 8, Table 1) differed 18.8 mm at maximum for a dorsal shift of the skull of 20 mm. This means that patient motion parallel to the scanning direction of the fan-beam-detector unit, heavily influence distances parallel to this direction.

**Table 1:** Mean differences of typical distances between reference points plus maximum deviation from reference image. The motion patterns are defined in Tab. 1. SD: standard deviation

Distance	Mean ± SD [mm]	Min[mm]	Max [mm]	Maximum difference to reference [mm]	Motion for image with maximum deviation
S–N	64.4 ± 5.2	60.8	82.8	−18.8	dorsal shift 20 mm
N-Me	112.3 ± 0.3	111.7	112.7	0.7	permanent nodding 5 mm

Regarding the cephalometric angles ANB angle (Ceph0: 7.5°) turned out to be a little less affected by patient motion than Maxillo-Mandibular Plane Angle (ANS-PNS to Me-Go) (Ceph0: 74.8°) (Fig. 9).

**Fig. 10:**
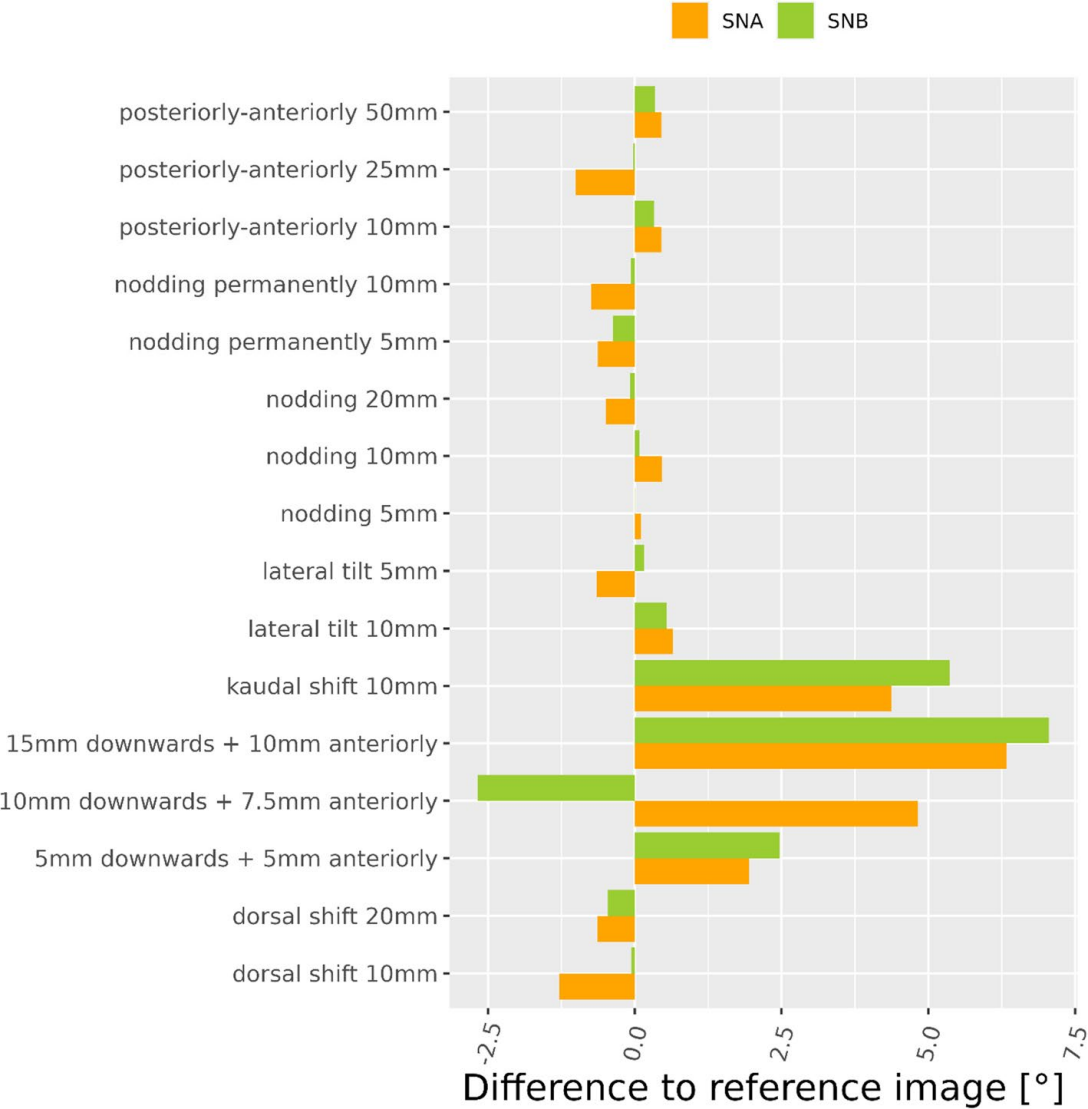
Angular deviations from reference image Ceph0 for SNA and SNB-angle

A larger effect was observed for SNA (Ceph0: 89.9°) and SNB (Ceph0: 82.4°). Particularly a downward shift of the head had a strong effect on the computed SNA and SNB angles (Fig. 10).

Analysis of variance revealed that the interaction of motion plus observer and also motion plus reference point highly significantly influenced the x- and y- coordinate definition (p < 0.001), whereas the observers as single factor had no significant influence on definition accuracy.

Patient motion, particularly parallel to the scanning direction of the fan-beam-detector unit, heavily influence distances parallel to this direction and, to a lesser extent, also angles relevant for orthodontic treatment planning.

## Discussion

Despite their certainly relative common use, there is only insufficient information published on the influence of patient motion on the location of cephalometric reference points within the resulting Ceph when using a vertically or horizontally scanning digital line-receptor. The latter are built in combined panoramic and cephalometric X-ray devices to reduce their costs since flat panel detectors are very costly [10].

Even to our big surprise, both inter-rater as well as intra-rater reproducibility was perfect in this study. To validate this finding, the intra-class-correlation was computed with two separate R-libraries (‘irr’ and ‘psych’), both with identical results (ICC = 1). This surprising finding is also supported by a mean difference between reading No. 1 and No. 2 over all readings of only 0.55 pixel (median = 0 pixel) for the x-coordinate versus a mean difference of 0.41 pixel (median = 0 pixel). We do not really have a good explanation for this excellent precision in detecting and marking the reference points. Maybe the lack of soft-tissues as scatter-material is one contributing factor as well as the fact, that only very well established, common reference points were marked. Other authors also found excellent agreement between and within raters [11, 12], supporting our observations.

The reason we used so many (20) observers was that we wanted to get reliable information about the location of each point by averaging all observers per point.

Our motion patterns and amplitudes were motivated by the measurements of Menzel and Gebauer [3]. The authors observed motion amplitudes of up to 15 mm in the vertical (sagittal) plane and up to 6 mm in the transversal plane. In anterior–posterior direction a maximum value of ca. 12 mm was measured. Obviously, some of our motion amplitudes even exceeded these values, yet this was applied to achieve a sufficient overview over different patterns and amplitudes that will likely occur in a clinical setting.

When comparing the location of the reference points between a static and a motion-beset Ceph, we observed significant differences in the location of the reference points especially for motion patterns parallel to the scanning direction. Hence, the horizontal distance S–N varied a lot with a maximum difference of 18.8 mm. As expected, the dorsal shift of the head (i.e. in the direction of the scanning motion of the image-receptor) resulted in the shortest distance S–N (Fig. 10).

However, such a motion is very much limited by the ear rods placed bilaterally in the patients’ ear canals. Yet these will allow for more rotational freedom within the vertical (sagittal) plane [13]. This also complies with the observations from Menzel and Gebauer [3]. Hence the horizontal scanning direction should be expected to be superior over a vertically scanning direction. This observation should be investigated further with a similar set-up in a vertically scanning device. In addition, clinicians should consider the use of a chin rest to further reduce the vertical movement of the head.

The angles describing sagittal and vertical jaw relationships were both rather stable over all motion patterns with maximum differences of 2° for the Maxillo-Mandibular Plane Angle versus 1.6° for the ANB angle. The relatively large ANB angle of 7.5° for Ceph0 indicates a skeletal Class II [14]. A motion-induced difference of ca. 2° in the ANB angle of a Class-II-patient might per se not influence orthodontic treatment planning, as the latter depends not only on a single cephalometric measurement but also on the type of malocclusion, facial type, soft tissue characteristics etc. However, the dramatic effect of motion on the horizontal distance S–N makes the affected cephalometric radiographs not suitable for evaluation of the anteroposterior position of the jaws (i.e. SNA, SNB angles) and for the analysis of growth or/and treatment changes over time as performed by superimposition techniques.

Therefore, devices with high scanning speeds give less time for patients to move their heads and should be preferred. In addition, technical progress in the future should focus on shorter scanning times of less than one second.

Even small differences in the ANB angle or the Maxillo-Mandibular Plane Angle can have a major impact as to whether a borderline patient qualifies for state coverage of the costs of the orthodontic treatment, as for example in the Swiss social insurance system [3]. Therefore, in patients with borderline cases, radiographs affected by movement should not be utilised for the final treatment planning. In such cases, the re-taking of the X-ray using a one-shot technique can be considered.

Overall, a one-shot cephalometric radiograph that does not allow for patient movement should be favoured over a scanned radiograph whenever feasible.

Using artificial intelligence to correct motion-induced distortions is a conceivable approach; however, the conceptual framework remains underdeveloped. Although a posteriori motion correction would be a very desirable option, from a physical perspective this seems very challenging due to the inherent ambiguity in 2D-projection radiography. The latter is well-known not to contain 3D-information of the object under study in a single image [15, 16]. Thus, additional information (e.g. photographic motion monitoring by means of stereo-cameras) augment the image information will be required to develop some sort of correction algorithm.

Future research could investigate the extent to which motion-induced distortions occur in scanned Cephs in comparison to one-shot Cephs taken in real patients.

## Conclusions

From our observations from assessment of typical cephalometric reference points we conclude, that patient motion, particularly parallel to the scanning direction of the fan-beam-detector unit, heavily influence clinically relevant distances parallel to this direction. Therefore, we recommend to use a horizontal scanning direction, to minimise scanning time to a minimum, or to prefer a one-shot technique if possible. Future advancements in this field may include the integration of artificial intelligence or algorithms for the purpose of motion correction.

## Supplementary Information

Supplementary Material 1.

Supplementary Material 2.

## Data Availability

No datasets were generated or analysed during the current study.

## Abbreviation

Ceph: Cephalometric radiograph
