# Supplementary material for: Influence of patient motion on definition of typical cephalometric reference points in digital horizontally scanning cephalometric radiography
Source: Head Face Med. 2025 Mar 17;21:18. doi: 10.1186/s13005-025-00491-z (PMC11912588; doi:10.1186/s13005-025-00491-z)
Supplement: Supplementary file 2 — Supplementary Material 2. [file 13005_2025_491_MOESM2_ESM.docx]

Supplementary materials

Stewart platform:

In general, a Stewart platform consists of two flat frames, the base, and the platform, which are connected by six linear actuators. By extending and retracting the individual actuators, the position and tilt of the upper platform can be adjusted. With a specially developed software component, it is possible to execute certain predefined movement patterns. The head mounting was achieved by means of an 3D-printed spine-replacement (material Tough-PLA (Poly Lactic Acid)) which was fixed through the foramen magnum.

Orthophos XG plus DS/Ceph

This machine integrates a vertically orientated ccd-line-detector (230 mm × 6.48 mm) which in combination with the vertical fan-beam is moved horizontally from anterior to posterior through the acquisition area with a speed of V(d) = 17 cm/14 s = 0.012 m/s. Consequently, the Ceph-image is acquired sequentially from the patient’s forehead to the back. The acquisition time for this study was 9.4 s per Ceph, pixel size amounted to 0.0945 x 0.0945 mm^2^. Images were exported as uncompressed DICOM-files.

Motion patterns used for evaluation:

| **Ceph No** | **Motion Pattern** |
| --- | --- |
| 1 | dorsal shift 10 mm |
| 2 | dorsal shift 20 mm |
| 3 | dorsal shift 100 mm (this Ceph was later omitted) |
| 4 | 5 mm downwards + 1 mm anterior shift |
| 5 | 10 mm downwards + 7.5 mm anterior shift |
| 6 | 15 mm downwards + 10 mm anterior shift |
| 7 | kaudal shift 10 mm |
| 8 | lateral tilt 10 mm |
| 9 | lateral tilt 5 mm |
| 10 | single nodding 10 mm |
| 11 | single nodding 20 mm |
| 12 | single nodding 5 mm |
| 13 | permanent nodding 5 mm |
| 14 | permanent nodding 10 mm |
| 15 | posterior-anterior shift 10 mm |
| 16 | posterior-anterior shift 25 mm |
| 17 | posterior-anterior shift 50 mm |
| 18 | steady reference Image (Ceph0) |

Millimeter values refer to motion amplitude.

Definition of the cephalometric points:

**S** *Sella Turcica:* The center of the pituitary fossa of the sphenoid bone (determined by inspection).

**N** *Nasion:* The junction of the frontonasal suture at the most posterior point on the curve at the bridge of the nose.

**ANS** *Anterior Nasal Spine:* The tip of the median, sharp bony process of the maxilla at the lower margin of the anterior nasal opening.

**A** *A Point:* The most posterior point on the curve of the maxilla between the anterior nasal spine and Supradentale.

**B** *B point:* The most posterior point on the anterior surface of the symphyseal outline of the mandible between Pogonion and Infradentale. Point B should lie within the apical third of the incisor roots.

**Pog** *Pogonion:* The most anterior point on the contour of the bony chin (determined by a tangent through Nasion).

**Me** *Menton:* The most inferior point on the symphyseal outline.

**Go** *Gonion:* The midpoint of the angle of the mandible. It is found by bisecting the angle formed by the ramus plane and the mandibular plane. The middle point of the right and left points is used.

**Ar** *Articulare:* The point of intersection of the inferior cranial base surface and the averaged posterior surfaces of the mandibular condyles.

**PNS** *Posterior Nasal Spine:* The most posterior point of the bony hard palate (the point of intersection of the hard palate and the pterygopalatine fossa).
